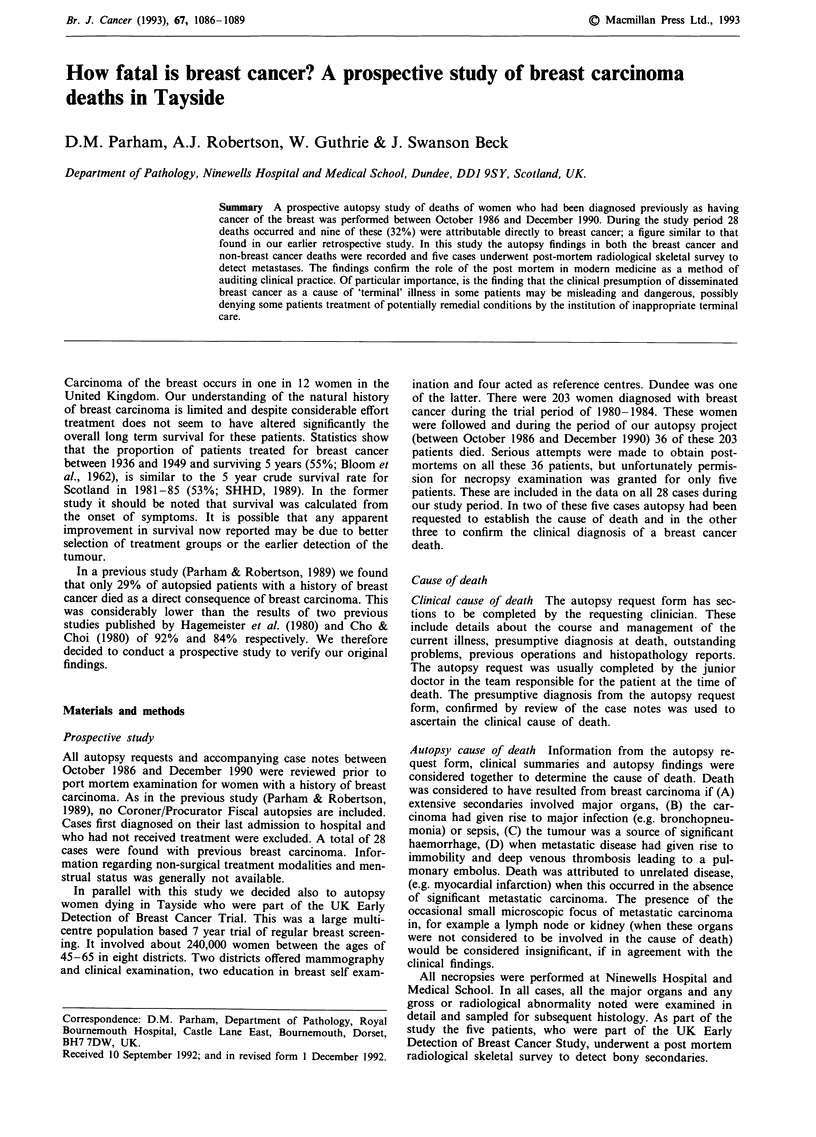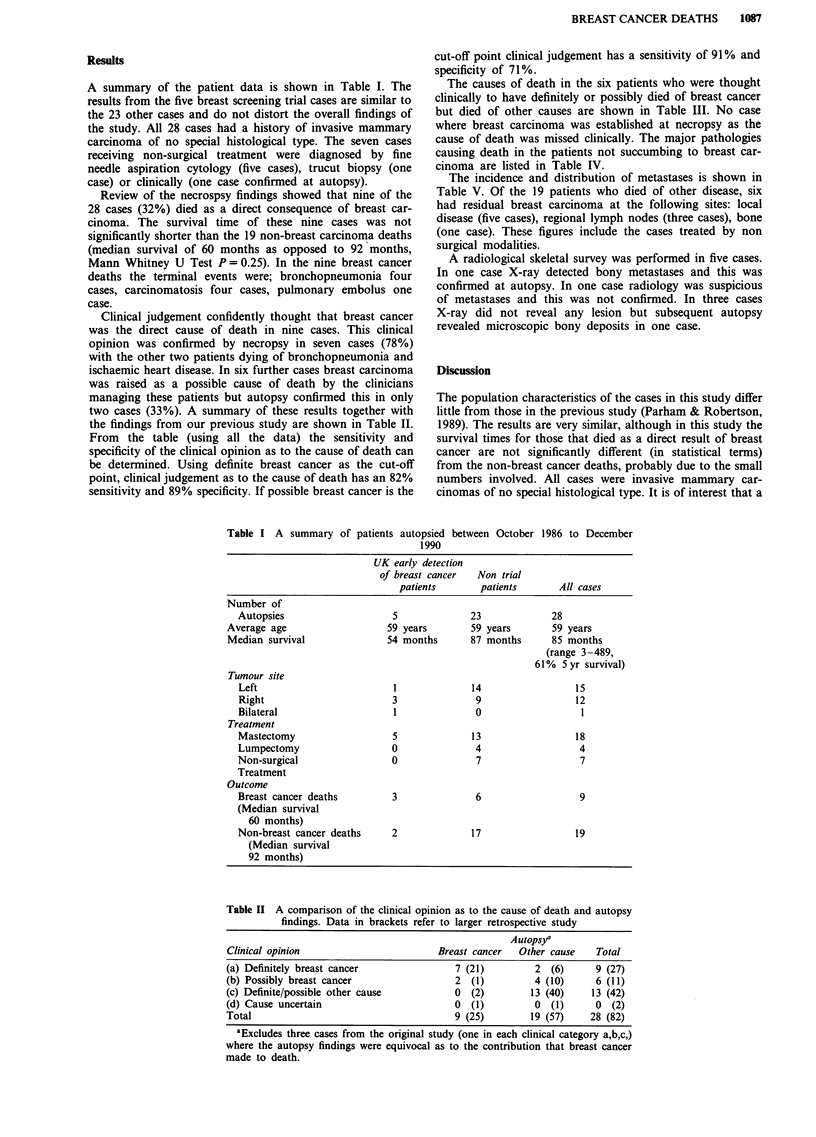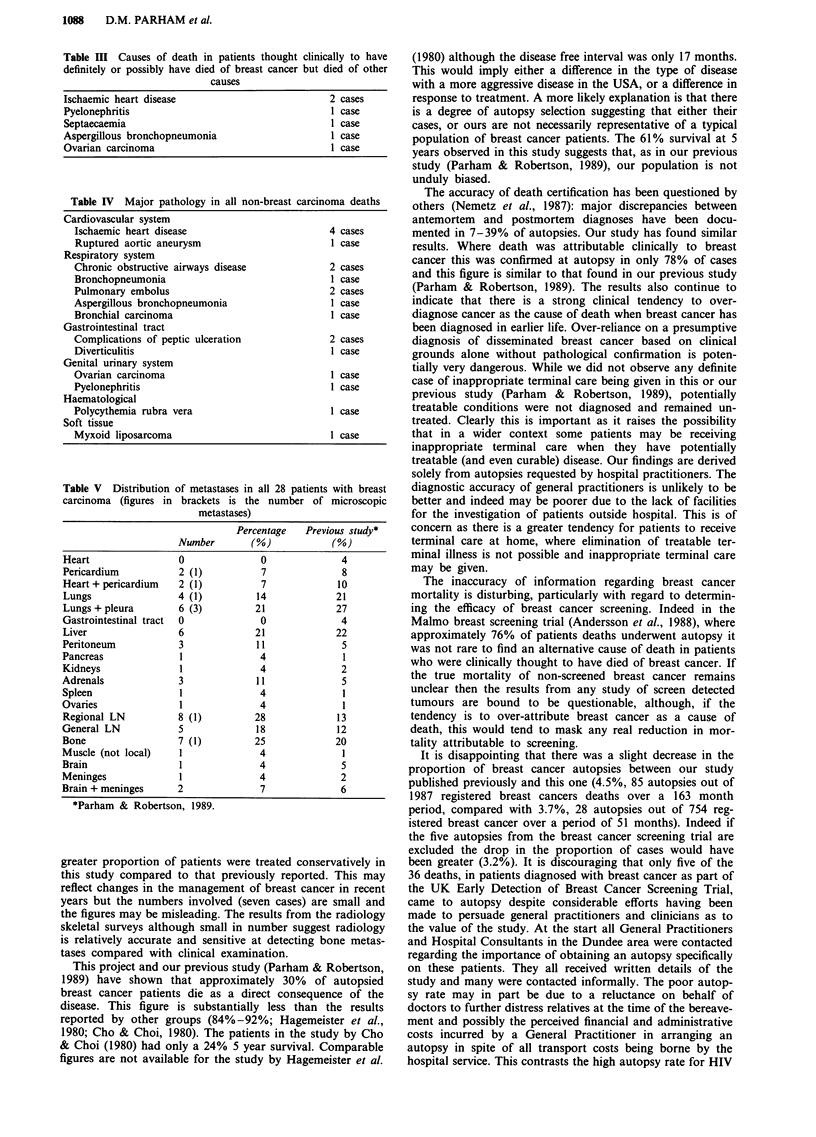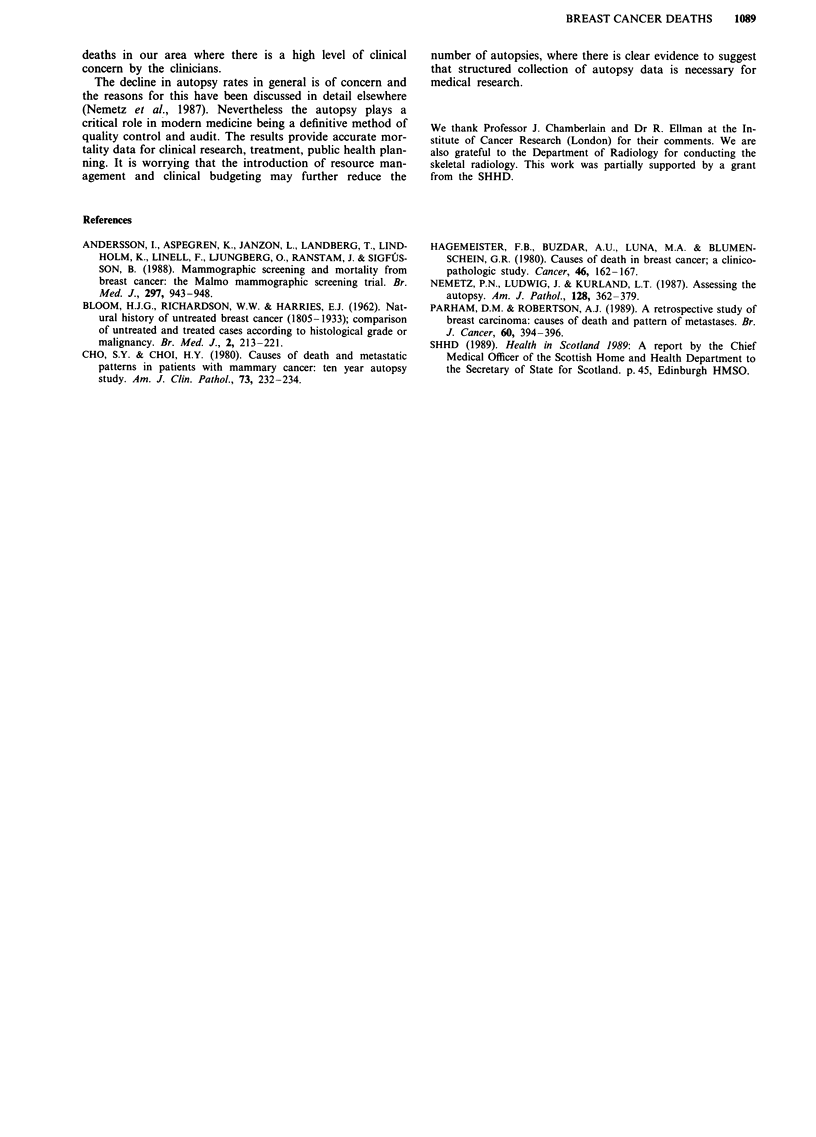# How fatal is breast cancer? A prospective study of breast carcinoma deaths in Tayside.

**DOI:** 10.1038/bjc.1993.199

**Published:** 1993-05

**Authors:** D. M. Parham, A. J. Robertson, W. Guthrie, J. S. Beck

**Affiliations:** Department of Pathology, Ninewells Hospital and Medical School, Dundee, Scotland, UK.

## Abstract

A prospective autopsy study of deaths of women who had been diagnosed previously as having cancer of the breast was performed between October 1986 and December 1990. During the study period 28 deaths occurred and nine of these (32%) were attributable directly to breast cancer; a figure similar to that found in our earlier retrospective study. In this study the autopsy findings in both the breast cancer and non-breast cancer deaths were recorded and five cases underwent post-mortem radiological skeletal survey to detect metastases. The findings confirm the role of the post mortem in modern medicine as a method of auditing clinical practice. Of particular importance, is the finding that the clinical presumption of disseminated breast cancer as a cause of 'terminal' illness in some patients may be misleading and dangerous, possibly denying some patients treatment of potentially remedial conditions by the institution of inappropriate terminal care.


 © Macmillan Press Ltd., 1993

### How fatal is breast cancer? A prospective study of breast carcinoma deaths in Tayside

D.M. Parham, A.J. Robertson, W. Guthrie & J. Swanson Beck

*Department of Pathology, Ninewells Hospital and Medical School, Dundee, DD1 9SY, Scotland, UK.*

**Summary** A prospective autopsy study of deaths of women who had been diagnosed previously as having cancer of the breast was performed between October 1986 and December 1990. During the study period 28 deaths occurred and nine of these (32%) were attributable directly to breast cancer; a figure similar to that found in our earlier retrospective study. In this study the autopsy findings in both the breast cancer and non-breast cancer deaths were recorded and five cases underwent post-mortem radiological skeletal survey to detect metastases. The findings confirm the role of the post mortem in modern medicine as a method of auditing clinical practice. Of particular importance, is the finding that the clinical presumption of disseminated breast cancer as a cause of 'terminal' illness in some patients may be misleading and dangerous, possibly denying some patients treatment of potentially remedial conditions by the institution of inappropriate terminal care.

Carcinoma of the breast occurs in one in 12 women in the United Kingdom. Our understanding of the natural history of breast carcinoma is limited and despite considerable effort treatment does not seem to have altered significantly the overall long term survival for these patients. Statistics show that the proportion of patients treated for breast cancer between 1936 and 1949 and surviving 5 years (55%; Bloom *et al.*, 1962), is similar to the 5 year crude survival rate for Scotland in 1981-85 (53%; SHHD, 1989). In the former study it should be noted that survival was calculated from the onset of symptoms. It is possible that any apparent improvement in survival now reported may be due to better selection of treatment groups or the earlier detection of the tumour.

In a previous study (Parham & Robertson, 1989) we found that only 29% of autopsied patients with a history of breast cancer died as a direct consequence of breast carcinoma. This was considerably lower than the results of two previous studies published by Hagemeister *et al.* (1980) and Cho & Choi (1980) of 92% and 84% respectively. We therefore decided to conduct a prospective study to verify our original findings.

### Materials and methods

### *Prospective study*

All autopsy requests and accompanying case notes between October 1986 and December 1990 were reviewed prior to port mortem examination for women with a history of breast carcinoma. As in the previous study (Parham & Robertson, 1989), no Coroner/Procurator Fiscal autopsies are included. Cases first diagnosed on their last admission to hospital and who had not received treatment were excluded. A total of 28 cases were found with previous breast carcinoma. Information regarding non-surgical treatment modalities and menstrual status was generally not available.

In parallel with this study we decided also to autopsy women dying in Tayside who were part of the UK Early Detection of Breast Cancer Trial. This was a large multicentre population based 7 year trial of regular breast screening. It involved about 240,000 women between the ages of 45-65 in eight districts. Two districts offered mammography and clinical examination, two education in breast self examination and four acted as reference centres. Dundee was one of the latter. There were 203 women diagnosed with breast cancer during the trial period of 1980-1984. These women were followed and during the period of our autopsy project (between October 1986 and December 1990) 36 of these 203 patients died. Serious attempts were made to obtain post-mortems on all these 36 patients, but unfortunately permission for necropsy examination was granted for only five patients. These are included in the data on all 28 cases during our study period. In two of these five cases autopsy had been requested to establish the cause of death and in the other three to confirm the clinical diagnosis of a breast cancer death.

### *Cause of death*

*Clinical cause of death* The autopsy request form has sections to be completed by the requesting clinician. These include details about the course and management of the current illness, presumptive diagnosis at death, outstanding problems, previous operations and histopathology reports. The autopsy request was usually completed by the junior doctor in the team responsible for the patient at the time of death. The presumptive diagnosis from the autopsy request form, confirmed by review of the case notes was used to ascertain the clinical cause of death.

*Autopsy cause of death* Information from the autopsy request form, clinical summaries and autopsy findings were considered together to determine the cause of death. Death was considered to have resulted from breast carcinoma if (A) extensive secondaries involved major organs, (B) the carcinoma had given rise to major infection (e.g. bronchopneumonia) or sepsis, (C) the tumour was a source of significant haemorrhage, (D) when metastatic disease had given rise to immobility and deep venous thrombosis leading to a pulmonary embolus. Death was attributed to unrelated disease, (e.g. myocardial infarction) when this occurred in the absence of significant metastatic carcinoma. The presence of the occasional small microscopic focus of metastatic carcinoma in, for example a lymph node or kidney (when these organs were not considered to be involved in the cause of death) would be considered insignificant, if in agreement with the clinical findings.

All necropsies were performed at Ninewells Hospital and Medical School. In all cases, all the major organs and any gross or radiological abnormality noted were examined in detail and sampled for subsequent histology. As part of the study the five patients, who were part of the UK Early Detection of Breast Cancer Study, underwent a post mortem radiological skeletal survey to detect bony secondaries.

Correspondence: D.M. Parham, Department of Pathology, Royal Bournemouth Hospital, Castle Lane East, Bournemouth, Dorset, BH7 7DW, UK.
Received 10 September 1992; and in revised form 1 December 1992.

### Results

A summary of the patient data is shown in Table I. The results from the five breast screening trial cases are similar to the 23 other cases and do not distort the overall findings of the study. All 28 cases had a history of invasive mammary carcinoma of no special histological type. The seven cases receiving non-surgical treatment were diagnosed by fine needle aspiration cytology (five cases), trucut biopsy (one case) or clinically (one case confirmed at autopsy).

Review of the necrospsy findings showed that nine of the 28 cases (32%) died as a direct consequence of breast carcinoma. The survival time of these nine cases was not significantly shorter than the 19 non-breast carcinoma deaths (median survival of 60 months as opposed to 92 months, Mann Whitney U Test $P = 0.25$). In the nine breast cancer deaths the terminal events were; bronchopneumonia four cases, carcinomatosis four cases, pulmonary embolus one case.

Clinical judgement confidently thought that breast cancer was the direct cause of death in nine cases. This clinical opinion was confirmed by necropsy in seven cases (78%) with the other two patients dying of bronchopneumonia and ischaemic heart disease. In six further cases breast carcinoma was raised as a possible cause of death by the clinicians managing these patients but autopsy confirmed this in only two cases (33%). A summary of these results together with the findings from our previous study are shown in Table II. From the table (using all the data) the sensitivity and specificity of the clinical opinion as to the cause of death can be determined. Using definite breast cancer as the cut-off point, clinical judgement as to the cause of death has an 82% sensitivity and 89% specificity. If possible breast cancer is the cut-off point clinical judgement has a sensitivity of 91% and specificity of 71%.

The causes of death in the six patients who were thought clinically to have definitely or possibly died of breast cancer but died of other causes are shown in Table III. No case where breast carcinoma was established at necropsy as the cause of death was missed clinically. The major pathologies causing death in the patients not succumbing to breast carcinoma are listed in Table IV.

The incidence and distribution of metastases is shown in Table V. Of the 19 patients who died of other disease, six had residual breast carcinoma at the following sites: local disease (five cases), regional lymph nodes (three cases), bone (one case). These figures include the cases treated by non surgical modalities.

A radiological skeletal survey was performed in five cases. In one case X-ray detected bony metastases and this was confirmed at autopsy. In one case radiology was suspicious of metastases and this was not confirmed. In three cases X-ray did not reveal any lesion but subsequent autopsy revealed microscopic bony deposits in one case.

### Discussion

The population characteristics of the cases in this study differ little from those in the previous study (Parham & Robertson, 1989). The results are very similar, although in this study the survival times for those that died as a direct result of breast cancer are not significantly different (in statistical terms) from the non-breast cancer deaths, probably due to the small numbers involved. All cases were invasive mammary carcinomas of no special histological type. It is of interest that a

**Table I** A summary of patients autopsied between October 1986 to December 1990

| | *UK early detection of breast cancer patients* | *Non trial patients* | *All cases* |
|---|---|---|---|
| Number of Autopsies | 5 | 23 | 28 |
| Average age | 59 years | 59 years | 59 years |
| Median survival | 54 months | 87 months | 85 months (range 3–489, 61% 5 yr survival) |
| *Tumour site* | | | |
| Left | 1 | 14 | 15 |
| Right | 3 | 9 | 12 |
| Bilateral | 1 | 0 | 1 |
| *Treatment* | | | |
| Mastectomy | 5 | 13 | 18 |
| Lumpectomy | 0 | 4 | 4 |
| Non-surgical Treatment | 0 | 7 | 7 |
| *Outcome* | | | |
| Breast cancer deaths (Median survival 60 months) | 3 | 6 | 9 |
| Non-breast cancer deaths (Median survival 92 months) | 2 | 17 | 19 |

**Table II** A comparison of the clinical opinion as to the cause of death and autopsy findings. Data in brackets refer to larger retrospective study

| | *Autopsy*[a] | | |
|---|---|---|---|
| *Clinical opinion* | *Breast cancer* | *Other cause* | *Total* |
| (a) Definitely breast cancer | 7 (21) | 2 (6) | 9 (27) |
| (b) Possibly breast cancer | 2 (1) | 4 (10) | 6 (11) |
| (c) Definite/possible other cause | 0 (2) | 13 (40) | 13 (42) |
| (d) Cause uncertain | 0 (1) | 0 (1) | 0 (2) |
| Total | 9 (25) | 19 (57) | 28 (82) |

[a]Excludes three cases from the original study (one in each clinical category a,b,c,) where the autopsy findings were equivocal as to the contribution that breast cancer made to death.

**Table III** Causes of death in patients thought clinically to have definitely or possibly have died of breast cancer but died of other causes

| | |
|---|---|
| Ischaemic heart disease | 2 cases |
| Pyelonephritis | 1 case |
| Septaecaemia | 1 case |
| Aspergillous bronchopneumonia | 1 case |
| Ovarian carcinoma | 1 case |

**Table IV** Major pathology in all non-breast carcinoma deaths

| | |
|---|---|
| Cardiovascular system | |
| Ischaemic heart disease | 4 cases |
| Ruptured aortic aneurysm | 1 case |
| Respiratory system | |
| Chronic obstructive airways disease | 2 cases |
| Bronchopneumonia | 1 case |
| Pulmonary embolus | 2 cases |
| Aspergillous bronchopneumonia | 1 case |
| Bronchial carcinoma | 1 case |
| Gastrointestinal tract | |
| Complications of peptic ulceration | 2 cases |
| Diverticulitis | 1 case |
| Genital urinary system | |
| Ovarian carcinoma | 1 case |
| Pyelonephritis | 1 case |
| Haematological | |
| Polycythemia rubra vera | 1 case |
| Soft tissue | |
| Myxoid liposarcoma | 1 case |

**Table V** Distribution of metastases in all 28 patients with breast carcinoma (figures in brackets is the number of microscopic metastases)

| | Number | Percentage (%) | Previous study* (%) |
|---|---|---|---|
| Heart | 0 | 0 | 4 |
| Pericardium | 2 (1) | 7 | 8 |
| Heart + pericardium | 2 (1) | 7 | 10 |
| Lungs | 4 (1) | 14 | 21 |
| Lungs + pleura | 6 (3) | 21 | 27 |
| Gastrointestinal tract | 0 | 0 | 4 |
| Liver | 6 | 21 | 22 |
| Peritoneum | 3 | 11 | 5 |
| Pancreas | 1 | 4 | 1 |
| Kidneys | 1 | 4 | 2 |
| Adrenals | 3 | 11 | 5 |
| Spleen | 1 | 4 | 1 |
| Ovaries | 1 | 4 | 1 |
| Regional LN | 8 (1) | 28 | 13 |
| General LN | 5 | 18 | 12 |
| Bone | 7 (1) | 25 | 20 |
| Muscle (not local) | 1 | 4 | 1 |
| Brain | 1 | 4 | 5 |
| Meninges | 1 | 4 | 2 |
| Brain + meninges | 2 | 7 | 6 |

*Parham & Robertson, 1989.

greater proportion of patients were treated conservatively in this study compared to that previously reported. This may reflect changes in the management of breast cancer in recent years but the numbers involved (seven cases) are small and the figures may be misleading. The results from the radiology skeletal surveys although small in number suggest radiology is relatively accurate and sensitive at detecting bone metastases compared with clinical examination.

This project and our previous study (Parham & Robertson, 1989) have shown that approximately 30% of autopsied breast cancer patients die as a direct consequence of the disease. This figure is substantially less than the results reported by other groups (84%–92%; Hagemeister *et al.*, 1980; Cho & Choi, 1980). The patients in the study by Cho & Choi (1980) had only a 24% 5 year survival. Comparable figures are not available for the study by Hagemeister *et al.* (1980) although the disease free interval was only 17 months. This would imply either a difference in the type of disease with a more aggressive disease in the USA, or a difference in response to treatment. A more likely explanation is that there is a degree of autopsy selection suggesting that either their cases, or ours are not necessarily representative of a typical population of breast cancer patients. The 61% survival at 5 years observed in this study suggests that, as in our previous study (Parham & Robertson, 1989), our population is not unduly biased.

The accuracy of death certification has been questioned by others (Nemetz *et al.*, 1987): major discrepancies between antemortem and postmortem diagnoses have been documented in 7–39% of autopsies. Our study has found similar results. Where death was attributable clinically to breast cancer this was confirmed at autopsy in only 78% of cases and this figure is similar to that found in our previous study (Parham & Robertson, 1989). The results also continue to indicate that there is a strong clinical tendency to overdiagnose cancer as the cause of death when breast cancer has been diagnosed in earlier life. Over-reliance on a presumptive diagnosis of disseminated breast cancer based on clinical grounds alone without pathological confirmation is potentially very dangerous. While we did not observe any definite case of inappropriate terminal care being given in this or our previous study (Parham & Robertson, 1989), potentially treatable conditions were not diagnosed and remained untreated. Clearly this is important as it raises the possibility that in a wider context some patients may be receiving inappropriate terminal care when they have potentially treatable (and even curable) disease. Our findings are derived solely from autopsies requested by hospital practitioners. The diagnostic accuracy of general practitioners is unlikely to be better and indeed may be poorer due to the lack of facilities for the investigation of patients outside hospital. This is of concern as there is a greater tendency for patients to receive terminal care at home, where elimination of treatable terminal illness is not possible and inappropriate terminal care may be given.

The inaccuracy of information regarding breast cancer mortality is disturbing, particularly with regard to determining the efficacy of breast cancer screening. Indeed in the Malmo breast screening trial (Andersson *et al.*, 1988), where approximately 76% of patients deaths underwent autopsy it was not rare to find an alternative cause of death in patients who were clinically thought to have died of breast cancer. If the true mortality of non-screened breast cancer remains unclear then the results from any study of screen detected tumours are bound to be questionable, although, if the tendency is to over-attribute breast cancer as a cause of death, this would tend to mask any real reduction in mortality attributable to screening.

It is disappointing that there was a slight decrease in the proportion of breast cancer autopsies between our study published previously and this one (4.5%, 85 autopsies out of 1987 registered breast cancers deaths over a 163 month period, compared with 3.7%, 28 autopsies out of 754 registered breast cancer over a period of 51 months). Indeed if the five autopsies from the breast cancer screening trial are excluded the drop in the proportion of cases would have been greater (3.2%). It is discouraging that only five of the 36 deaths, in patients diagnosed with breast cancer as part of the UK Early Detection of Breast Cancer Screening Trial, came to autopsy despite considerable efforts having been made to persuade general practitioners and clinicians as to the value of the study. At the start all General Practitioners and Hospital Consultants in the Dundee area were contacted regarding the importance of obtaining an autopsy specifically on these patients. They all received written details of the study and many were contacted informally. The poor autopsy rate may in part be due to a reluctance on behalf of doctors to further distress relatives at the time of the bereavement and possibly the perceived financial and administrative costs incurred by a General Practitioner in arranging an autopsy in spite of all transport costs being borne by the hospital service. This contrasts the high autopsy rate for HIV

deaths in our area where there is a high level of clinical concern by the clinicians.

The decline in autopsy rates in general is of concern and the reasons for this have been discussed in detail elsewhere (Nemetz *et al.*, 1987). Nevertheless the autopsy plays a critical role in modern medicine being a definitive method of quality control and audit. The results provide accurate mortality data for clinical research, treatment, public health planning. It is worrying that the introduction of resource management and clinical budgeting may further reduce the number of autopsies, where there is clear evidence to suggest that structured collection of autopsy data is necessary for medical research.

We thank Professor J. Chamberlain and Dr R. Ellman at the Institute of Cancer Research (London) for their comments. We are also grateful to the Department of Radiology for conducting the skeletal radiology. This work was partially supported by a grant from the SHHD.

### References

ANDERSSON, I., ASPEGREN, K., JANZON, L., LANDBERG, T., LINDHOLM, K., LINELL, F., LJUNGBERG, O., RANSTAM, J. & SIGFÚSSON, B. (1988). Mammographic screening and mortality from breast cancer: the Malmo mammographic screening trial. *Br. Med. J.*, **297**, 943–948.

BLOOM, H.J.G., RICHARDSON, W.W. & HARRIES, E.J. (1962). Natural history of untreated breast cancer (1805–1933); comparison of untreated and treated cases according to histological grade or malignancy. *Br. Med. J.*, **2**, 213–221.

CHO, S.Y. & CHOI, H.Y. (1980). Causes of death and metastatic patterns in patients with mammary cancer: ten year autopsy study. *Am. J. Clin. Pathol.*, **73**, 232–234.

HAGEMEISTER, F.B., BUZDAR, A.U., LUNA, M.A. & BLUMENSCHEIN, G.R. (1980). Causes of death in breast cancer; a clinicopathologic study. *Cancer*, **46**, 162–167.

NEMETZ, P.N., LUDWIG, J. & KURLAND, L.T. (1987). Assessing the autopsy. *Am. J. Pathol.*, **128**, 362–379.

PARHAM, D.M. & ROBERTSON, A.J. (1989). A retrospective study of breast carcinoma: causes of death and pattern of metastases. *Br. J. Cancer*, **60**, 394–396.

SHHD (1989). *Health in Scotland 1989*: A report by the Chief Medical Officer of the Scottish Home and Health Department to the Secretary of State for Scotland. p. 45, Edinburgh HMSO.